# A Hierarchical Planning Method for AUV Search Tasks Based on the Snake Optimization Algorithm

**DOI:** 10.3390/s24227196

**Published:** 2024-11-10

**Authors:** Zhiwen Wen, Zhong Wang, Xiangdong Wen, Chenxi Niu, Pei Wang, Daming Zhou

**Affiliations:** 1 Xi’an Precision Machinery Research Institute, Xi’an 710077, China; wenzhiwen0506@163.com (Z.W.);; 2 School of Astronautics, Northwestern Polytechnical University, Xi’an 710072, China

**Keywords:** autonomous underwater vehicles, search tasks, hierarchical programming approach, snake optimization algorithm

## Abstract

In a complex and dynamic battlefield environment, enabling autonomous underwater vehicles (AUVs) to reach dynamic targets in the shortest possible time using global autonomous planning is a key issue affecting the completion of search tasks. In this study, ahierarchicalAUV task planning method that uses a combination of hierarchical programming and a snake optimization algorithm is proposed for two typical cases where the platform can provide initial target information. This method decomposes the search task problem into a three-level programming problem, with the outer task planning goal of achieving the shortest encounter time between AUV and dynamic targets; the goal of task planning in the middle layer is to achieve the shortest actual navigation time for AUVs under different operating conditions; and the internal task planning is responsible for considering the comprehensive trajectory optimization under navigation constraints such as threat zone, path length, and path smoothness. The snake optimization algorithm was used for solving each layer of task planning. The feasibility of the proposed method was verified through simulation experiments of AUV search tasks under two types of initial target information conditions. The simulation results show that this method can achieve task planning for AUV searching for dynamic targets under various constraint conditions, optimize the encounter time between AUV and dynamic targets, and have strong engineering practical value. It has certain reference significance for task planning problems similar to underwater unmanned equipment.

## 1. Introduction

In recent years, with the rapid development of science and technology and the deepening understanding of the ocean, underwater space has become a new focus of international strategic competition. As a multiplier of naval power, the autonomous underwater vehicle (AUV) has seen unprecedented development in applications such as underwater reconnaissance, underwater communication, anti-submarine and anti-mine warfare, and information warfare. As a manifestation of AUV’s intelligence level, the capability of autonomous task planning significantly affects the success rate of AUV missions. Current global task planning methods mainly focus on solving planning problems for static environments or known navigation endpoints. However, in practical applications, due to the partial observability of the task environment and the dynamic changes in target information, the environmental information relied on for planning is uncertain. In fact, AUVs are more often used for searching and exploring dynamic targets. Before being launched by the platform, task planning can be divided into two categories based on the completeness of the initial dynamic target information obtained through platform detection: task planning under initially complete target information and task planning under initially incomplete target information. Initial complete target information refers to the relatively complete dynamic target information that can be obtained by the platform before deploying the AUV, including target location, distance, heading, and speed information. In contrast, initial incomplete target information refers to the situation where the platform can only obtain approximate dynamic target information before deploying the AUV, such as target location and approximate distance information. In light of these circumstances, the key issue that urgently needs to be solved is how to efficiently perform global task planning for the AUV based on limited platform target information before deploying the AUV, so that the AUV has a high degree of autonomy for locating dynamic targets.

The United States is the earliest country to research and equip combat aircraft mission planning systems and has experienced three generations of development [1,2]. Greshler et al. [3] introduced cooperative conflict-basedsearch (Co-CBS) to integrate the CBS algorithm based on a multi-agent collaboration framework, enabling collaborative task allocation and path planning for multiple robots. Other studies proposed amodified A-Star algorithm containing the bidirectional sector expansion and variable step search strategy for penetration path planning for stealth unmanned aerial vehicles (SUAVs) [4]. An innovative path planning algorithm that synergizes the A* algorithm with the rapidly-exploring random tree (RRT) approach was proposed to improve planning efficiency and path smoothness [5]. However, the above-mentioned path planning algorithm still has the disadvantage of easily falling into the local optimal solution.

To improve the efficiency of the solution, some scholars have utilized intelligence heuristic algorithms for task allocation, such as genetic algorithms [6], particle swarm optimization [7], and simulated annealing (SA) algorithms [8]. Yu Jing et al. [9] designed a double-layer mutually coupled task planning solution strategy that divides the task planning model into upper-level task allocation and lower-level task sequence optimization, with specific optimization methods and steps for each layer. 

In order to break through the inherent scalability limitations of traditional centralized control path planning algorithms, Guo et al. [10] designed a decentralized path planning algorithm predicated on deep reinforcement learning to enhance the flexibility, robustness, and scalability of automated guided vehicles (AGVs). Chen et al. [11] and Xiao et al. [12] described the task planning problem as a mixed-integer linear programming (MILP) model and solved it using a linear programming solver, which can yield stable and effective optimization results. In the literature [13], the strike task is decomposed into a multi-objective task allocation problem under time constraints and a trajectory planning problem, with the two sub-problems being mutually coupled. Wang Bo et al. [14] decomposed the planning task into an upper-level planning for designing intermediate configurations and a lower-level planning for solving the movement methods of achieving those intermediate configurations. Each level of planning is solved independently, reducing the uncertainty and complexity of the satellite reconstruction planning problem. Bai et al. [15] proposed a novel two-layer algorithm SA-reCBS that cascades the simulated annealing algorithm and conflict-based search to solvemulti-robot task assignment and path-finding problemswithout requiring a pre-bundle of tasks to groups with the same number of groups as the number of robots. Yang Han and Yu et al. [16] proposed a heuristic called Space Utilization Optimization (SUO) for path planning. This heuristic aims to significantly reduce conflicts among robots while maintaining the optimality of the paths. Chen Zihao et al. [17] proposed a cluster task planning method based on a double-layer optimization model. The lower-level optimization focuses on trajectory planning, while the upper-level optimization is task allocation, improving the optimality of the task planning results.

From top-level planning to layered and gradual implementation, this approach helps to reduce the complexity of the problem. In terms of task planning solutions, heuristic algorithms such as particle swarm optimization, differential evolution, wolf pack algorithm [18], genetic algorithms [19,20], ant colony optimization [21], and firefly algorithm [22] have received widespread attention from scholars in recent years due to their simplicity and effectiveness. Zhu et al. [23] introduced a biologically inspired integrated self-organizing map algorithm for task allocation and path planning of AUV systems in three-dimensional underwater obstacle avoidance environments. Zhang et al. [24] used the sine cosine algorithm to improve the Harris Hawk algorithm for path planning, enhancing its robustness. However, they were unable to estimate the convergence speed of the algorithm in complex environments. Yu et al. [25] proposed a traversal multi-target path planning method for multiple unmannedsurfacevehicles (USVs). The proposed algorithm consists of two parts: First, the improved Hungary algorithm is proposed for multi-target task allocation. Second, the virtual field sampling algorithm and the ocean current constraint function are introduced in the RRT* algorithm for path planning. Through the improvement of the algorithm, the efficiency of multi-target task allocation improves, and the time spent path planning is reduced. However, the algorithm’slow convergence accuracy remains a problem. Han. et al. [26] designed a full-dimensional ABC algorithm with an adaptive factor to enhance the search capability of AUVs. Although the improved algorithm solves the problem of planning global paths for AUVs to search for targets, it easily falls into the local optimum. Zou et al. [27] proposed a novel population-based iterated greedy (PIG) algorithm for multi-automated guided vehicles(multi-AGV)scheduling problemsinvolvingunloading safety detection. In the PIG, a hyper-heuristic based on neighborhood operators and a population-based initialization method are proposed to obtain multiple high-quality initial solutions. In another study, Han et al. [28] propose a novel mixed integer linear programming (MILP) model and a dual population collaborative genetic algorithm (DCGA). The integration problems of the flexible job shop scheduling problem (FJSP) and minimizing the makespan of AGVswere studied. Cui et al. [29] proposed a mixed-integer linear programming model and adiscrete Jaya (DJaya) algorithm to the multi-AGVs scheduling problem with dynamic unloading time. This algorithm consists of two parts: (1) A heuristic based on the ant colony algorithm is used in the initial solution; (2) two DJaya operators are designed for the best and worst solutions. The computational results show that the proposed DJaya algorithm is superior to the existing algorithms in tackling the considered problem.

Although the above modifications have improved efficiency, they cannot predict the convergence speed of algorithms in complex environments, and there is an imbalance between global exploration and local development. The above algorithms have higher optimization accuracy than traditional classical algorithms and show significant improvement in path planning and task allocation in unmanned systems. However, this does not necessarily mean that these algorithms have the best optimization accuracy and task planning quality. Their optimization accuracy and path planning quality in different scenarios still need to be improved. Attempting to improve different algorithms through different strategies for solving task planning problems in unmanned systems still has research significance.

The task planning problem for AUVs to track dynamic targets can be decomposed into two coupled sub-problems: the problem of being able to intercept the dynamic target and the problem of minimizing the time to interception. The aim of this paper is to propose an autonomous global task planning method for AUVs by combining two typical types of initial observed target information. 

This study proposes a hierarchical task planning method based on the snake algorithm, which hierarchically designs the task allocation problem of AUVs. A multi-layer task planning model is proposed for use in conjunction with the snake optimization algorithm to achieve multi-layer nested optimization and address the task planning problem of AUVs under typical target initial information conditions. Using MATLAB software, this study develops algorithm validation programs, constructs complex underwater task scenarios, sets initial scenarios for typical tasks, uses the designed algorithms for task planning, and verifies its application effectiveness.

The main innovations of this study are as follows: (1) The adoption of a three-level planning strategy to address the aforementioned problems, which makes it easier to obtain global optimal solutions for dynamic targets; (2) the introduction of a snake optimization algorithm into each level of task planning, enabling better objective function values; (3) the development of two different three-level task planning methods tailored to the two maintypes of initial target detection information provided by the platform, effectively addressing practical engineering problems.

This paper is organized as follows: Section 2 describes the snake optimization algorithm and hierarchical task planning method. Section 3 gives the experimental design and discusses the experimental results. Finally, Section 4 provides our conclusion.

## 2. Materials and Methods

### 2.1. Assumptions

For the purpose of this study, which focuses on AUV autonomous task planning, and in consideration of the practical working characteristics of AUVs, the following simplifications and assumptions are made:

1. The AUV is treated as a point mass within the vast underwater space.
2. Obstacles and threat areas are considered collision zones, and irregular collision zones are uniformly “inflated” to cylindrical shapes for simplicity.
3. Factors such as tidal currents, sea currents, electronic interference, and other disturbances are not considered.
4. During simulations, both the AUV and the dynamic target move with constant speeds.

### 2.2. Environment Modeling

The underwater three-dimensional space is modeled using a grid-based approach. This study considers a scenario where an underwater platform deploys an AUV to search for and monitor a dynamic target. After the underwater platform acquires some initial target information, it releases the AUV to conduct a close-range search and surveillance of the dynamic target.

**Figure 1:**
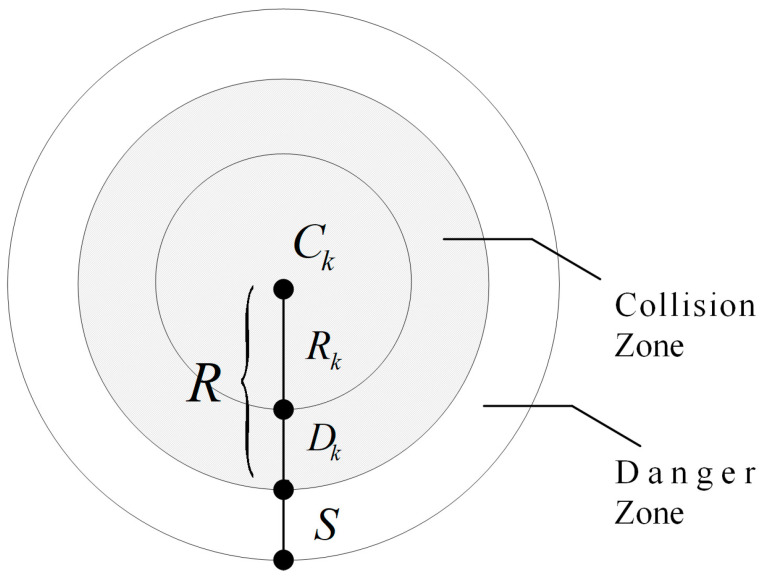
Obstacle symbol description.

In Figure 1, we assume that each obstacle or threat is defined within a cylinder with a projection that has a center coordinate $C_{k}=\left[x,y,z\right]$ and a radius $R_{k}$. $R_{k}$ is the maximum distance between the surface and the center of an irregular sphere. $D_{k}$ represents the AUV safety margin distance and S is the critical distance from the AUV to the collision zone, which depends on the specific application, operating environment, and positioning accuracy. In this simulation, R is the radius of the cylinder, which is identified as an obstacle or threat and is defined as $R=R_{k}+D_{k}$. Here, based on the size of the AUV, select $D_{k}=20\;m$. x and  y represent the center coordinates of the obstacle, while z represents its height. In practical applications, the terrain height needs to be added to *z* for processing. The obstacle data used in the simulation are presented in Table 1.

**Figure 2:**
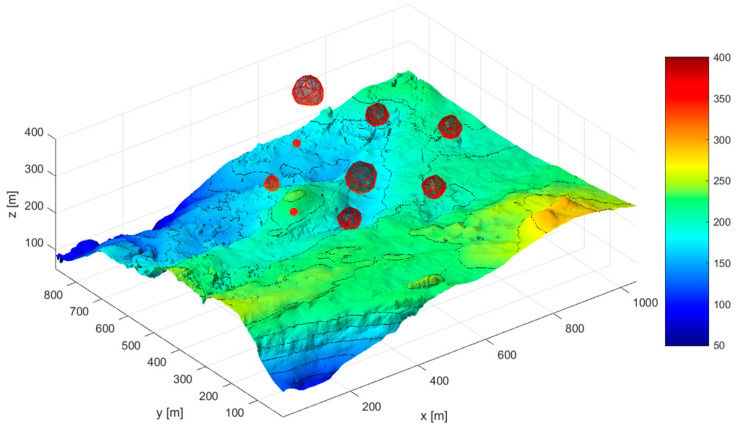
Submarine model after adding obstacles.

The environment design program was developed in MATLAB 2018b. We obtained seabed elevation information by loading elevation image files in tif format, generating seabed terrain grid coordinates using the meshgrid command, and establishingthe seabed terrain. We set the obstacle position in Matlab, using the meshgird() command to generate the surface coordinates of the obstacle, using the surf() command to smooth and generate irregular obstacles, and taking the maximum distance from the center as the radius of the obstacle. After displaying obstacles on the underwater terrain image, we completed the environment modeling. The environment model is shown in Figure 2. Several obstacles of different sizes and radiiwere added to the constructed seabed model to simulate the threat areas that the AUV may encounter during navigation.

### 2.3. Snake Optimization Algorithm

**Figure 3:**
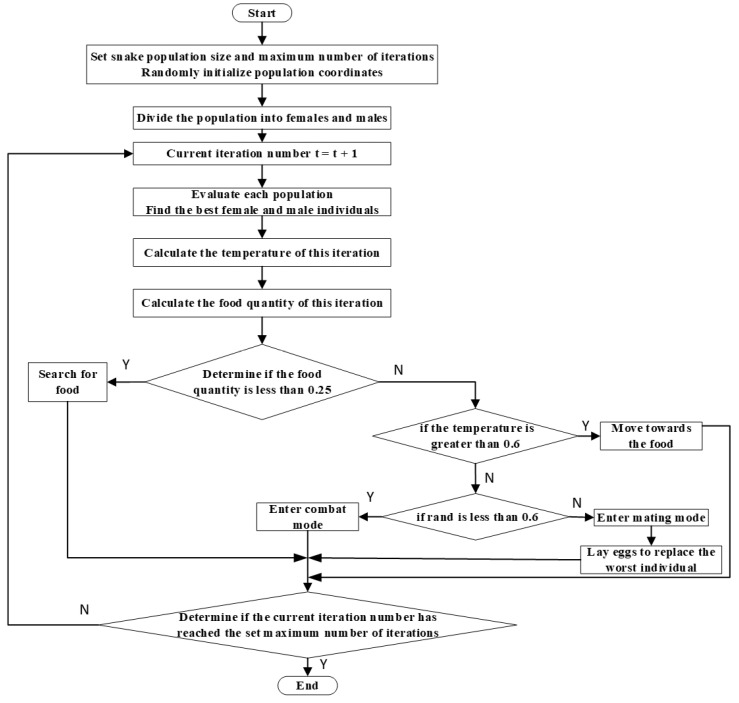
SO algorithm flowchart. (“N” = “No”, “Y” = “Yes”, “t” = “iteration number”, subsequent settings are the same here).

Inspired by the unique mating behavior of snakes, Fatma A. Hashim and Abdelazim G. Hussien proposed the snake optimizer (SO) algorithm in 2022 [30]. The SO algorithm is a new meta-heuristic optimization algorithm that simulates the different behavioral patterns of snakes under different temperatures and food conditions to search for the optimal value. The flow of the SO algorithm is shown in Figure 3. The definitions of the parameters involved in the SO algorithm are shown in Table 2.

First, the snake population is initialized:

(1) $$X_{i}=X_{i,min}+\mathrm{rand}\;\times \;(X_{i,max}-X_{i,min}),$$

where $X_{i}$ represents the position of the ith individual, rand is a random number between 0 and 1, and $X_{i,min}$ and $X_{i,max}$ are the lower and upper bounds of the problem, respectively.

After the snake population is randomly initialized in the search space, the population is evenly divided into two groups: male and female. The number of males and females is represented by the following two formulas, respectively:

(2) $$\begin{array}{c} N_{m}\approx \frac{N}{2}\\ N_{f}=N-N_{m} \end{array},$$

where N represents the total population size, *Nm* is the number of male individuals, and *Nf* is the number of female individuals. From each group, the best individual is identified to obtain the positions of the best male, best female, and food, denoted as *f*_*best,m*_, *f*_*best,f*_, and *f*_*food*_, respectively. The definition of temperature is as follows:

(3) $$Temp=\exp\left(\frac{-t}{T}\right),$$

where t represents the current iteration number and T represents the maximum iteration number. The amount of food is defined as:

(4) $$Q=c_{1}\times \exp\left(\frac{t-T}{T}\right),$$

where c1 is a constant, taken as 0.5.

The exploration phase describes the environmental factors of cold regions and food, where snakes do not actively search for food in their immediate surroundings. If Q < Threshold (where Threshold = 0.25), snake individuals search for food by randomly selecting positions and updating their own positions accordingly. The exploration phase of the snake population is simulated as follows:

(5) $$X_{i,m\left(f\right)}\left(t+1\right)=X_{\mathrm{rand},m\left(f\right)}\left(t\right)\pm c_{2}\times A_{m\left(f\right)}\times ((X_{max}-X_{min})\times \mathrm{rand})+X_{max},$$

In the formula: *Xi*,*m*(*f*) represents a male or female individual, *rand* is a random number in the range [0,1]; *c*2 is taken as 0.05; and *Am*(*f*) represents the ability of males or females to search for food, which is given by the following formula:

(6) $$A_{m\left(f\right)}=\exp\left(\frac{-f_{rand,m\left(f\right)}}{f_{i,m\left(f\right)}}\right),$$

where *f*_*rand*,*m*(*f*)_ is the fitness value of the randomly selected male or female individual position *X*_*rand*,*m*(*f*)_, and *f*_*i*,*m*(*f*)_ is the fitness value of the ith individual position *X*_*i*,*m*(*f*)_ in the male or female population.

During the exploitation phase, there is food available, i.e., *Q* > *Threshold*. If the temperature is high, represented by *temperature* > *Threshold* (0.6), snakes will only search for food, and the position update formula is as follows:

(7) $$X_{i,j}\left(t+1\right)=X_{food}\pm c_{3}\times \mathrm{Temp}\times \mathrm{rand}\times \left(X_{food}-X_{i,j}\left(t\right)\right),$$

where *X*_*i*,*j*(*t*)_ represents the individual’s position and the best position for a snake individual is *X*_*food*_, with *c*_3_ taking the value of 2.

If the temperature is below the threshold, i.e., *temperature* < *Thresgold*, indicating a cold environment, individual snake will enter either combat or mating mode. The position update formula in combat mode is as follows:

(8) $$X_{i,m\left(f\right)}\left(t+1\right)=X_{i,m\left(f\right)}\left(t\right)\pm c_{3}\times FM\left(F\right)\times rand\times \left(Q\times X_{best,f\left(m\right)}-X_{i,m\left(f\right)}\left(t\right)\right)$$

In the formula, *Xbest*, *f*(*m*) represents the best position in the female (male) group, and *FM*(*F*) represents the fighting ability of males or females. It is calculated as follows:

(9) $$FM\left(F\right)=\exp\left(\frac{-f_{best,f\left(m\right)}}{f_{i}}\right),$$

In the formula, *f*_*best*,*f*(*m*)_ represents the fitness value of the best male or female individual, while the current *f*_*i*_ is the fitness value of a snake individual.

In mating mode, simply replace *X*_*best*,*f*(*m*)_ with *X*_*i*,*f*(*m*)_, which represents the position of the ith female or male individual. Additionally, replace *FM*(*F*) with *M*_*m*(*f*)_ to represent the mating ability of males and females. It can be calculated as follows:

(10) $$M_{m\left(f\right)}=\exp\left(\frac{-f_{i,f\left(m\right)}}{f_{i,m\left(f\right)}}\right),$$

If snake eggs hatch, the male and female with the worst fitness are selected to replace their positions, which can be represented as:

(11) $$X_{\mathrm{worst},m\left(f\right)}=X_{min}+\mathrm{rand}\times \left(X_{max}-X_{min}\right),$$

The pseudocode of the SO algorithm is in Algorithm 1.
**Algorithm 1.** Snake Optimization Algorithm$\begin{array}{l} \mathrm{Input}\text{:}\mathrm{pop},\mathrm{Iteration},\mathrm{fobj},\;\dim,X\text{\_}\min,X\text{\_}\max\\ \mathrm{Output}\text{:}\mathrm{Xfood},\mathrm{fval},\mathrm{gbest}\text{\_}t\\ 1{.\;\mathrm{Initialize}\;\mathrm{ThresholdQ}\;\mathrm{ThresholdT},c}_{1}{,c}_{2}{,c}_{3}\\ 2.\;X=\mathrm{initializtion}(\mathrm{pop},\dim,X\text{\_}\max,X\text{\_}\min)\\ 3.\;\mathrm{Calculate}\;\mathrm{fobj}\;\mathrm{function}\;\mathrm{fitness}\;\mathrm{value}\;\mathrm{for}\;\mathrm{each}\;X\\ 4.\;[\mathrm{GYbest},\mathrm{gbest}]=\min(\mathrm{fitness}),\mathrm{Xfood}=X(\mathrm{gbest},\text{:})\\ 5.\;\mathrm{Nm}=\mathrm{round}(\mathrm{pop}/2),\;\mathrm{Nf}=\mathrm{pop}-\mathrm{Nm}\\ 6.\;\mathrm{Xm}=X(1\text{:}\mathrm{Nm},\text{:}),\;\mathrm{Xf}=X(\mathrm{Nm}+1\text{:}\mathrm{pop},\text{:})\\ 7.\;\mathrm{fitness}\text{\_}m=\mathrm{fitness}(1\text{:}\mathrm{Nm}),\;\mathrm{bestX}\text{\_}m=\mathrm{bestX}(1\text{:}\mathrm{Nm},\text{:})\;\%\mathrm{Male}\;\mathrm{snake}\;\mathrm{population}\;\\ 8.\;[\mathrm{fitnessBest}\text{\_}m,\mathrm{gbest}1]=\min(\mathrm{fitness}\text{\_}m),\;\mathrm{Xbest}\text{\_}m=\mathrm{Xm}(\mathrm{gbest}1,\text{:})\\ 9.\;\mathrm{fitness}\text{\_}f=\mathrm{fitness}(\mathrm{Nm}+1\text{:}\mathrm{pop}),\;\mathrm{bestX}\text{\_}f=\mathrm{bestX}(\mathrm{Nm}+1\text{:}\mathrm{pop},\text{:})\;\%\mathrm{Female}\;\mathrm{snake}\;\mathrm{population}\\ 10.\;[\mathrm{fitnessBest}\text{\_}f,\mathrm{gbest}2]=\min(\mathrm{fitness}\text{\_}f),\;\mathrm{Xbest}\text{\_}f=\mathrm{Xm}(\mathrm{gbest}2,\text{:})\\ 11.\;\mathrm{for}\;i=1\text{:}\mathrm{Iteration}\\ 12{.\;\;\mathrm{Temp}=\exp(-((t)/\mathrm{Iteration})),\;Q=c}_{1}\times \exp(((t-\mathrm{Iteration})/\mathrm{Iteration}))\\ 13.\;\;\;\mathrm{if}\;Q<\mathrm{Threshold}\\ 14.\;\;\;\;\;\;\mathrm{update}\;\mathrm{Xnewm}\;\mathrm{use}\;\mathrm{Xm}\;\mathrm{by}\;\mathrm{Equation}\;(5)\\ 15.\;\;\;\;\;\;\mathrm{update}\;\mathrm{Xnewf}\;\mathrm{use}\;\mathrm{Xf}\;\mathrm{by}\;\mathrm{Equation}\;(5)\;\\ 16.\;\;\;\mathrm{else}\\ 17.\;\;\;\;\;\mathrm{if}\;\mathrm{Temp}>\mathrm{Thresold}2\\ 18.\;\;\;\;\;\;\mathrm{update}\;\mathrm{Xnewm}\;\mathrm{use}\;\mathrm{Xbest}\;\mathrm{by}\;\mathrm{Equation}\;(7)\\ 19.\;\;\;\;\;\;\mathrm{update}\;\mathrm{Xnewf}\;\mathrm{use}\;\mathrm{Xbest}\;\mathrm{by}\;\mathrm{Equation}\;(7)\;\\ 20.\;\;\;\;\;\mathrm{else}\\ 21.\;\;\;\;\;\;\mathrm{if}\;\mathrm{rand}>0.6\\ 22.\;\;\;\;\;\;\;\mathrm{update}\;\mathrm{Xnewm}\;\mathrm{use}\;\mathrm{Xm}\;\mathrm{by}\;\mathrm{Equation}\;(8)\\ 23.\;\;\;\;\;\;\;\mathrm{update}\;\mathrm{Xnewf}\;\mathrm{use}\;\mathrm{Xf}\;\mathrm{by}\;\mathrm{Equation}\;(8)\\ 24.\;\;\;\;\;\;\mathrm{else}\\ 25.\;\;\;\;\;\;\;\mathrm{update}\;\mathrm{Xnewm}\;\mathrm{use}\;\mathrm{Xm}\;\mathrm{by}\;\mathrm{Equation}\;(8)\;\mathrm{replace}\;\mathrm{Fm}\;\mathrm{with}\;\mathrm{Mm}\;\\ 26.\;\;\;\;\;\;\;\mathrm{update}\;\mathrm{Xnewf}\;\mathrm{use}\;\mathrm{Xf}\;\mathrm{by}\;\mathrm{Equation}\;(8)\;\mathrm{replace}\;\mathrm{Fm}\;\mathrm{with}\;\mathrm{Mm}\;\\ 27.\;\;\;\;\;\;\;\;\mathrm{if}\;\mathrm{rand}>0.5\\ 28.\;\;\;\;\;\;\;\;\;\mathrm{update}\;\mathrm{Xnewm}\;\mathrm{wrost}\;\mathrm{by}\;\mathrm{Equation}\;(11)\\ 29.\;\;\;\;\;\;\;\;\;\mathrm{update}\;\mathrm{Xnewf}\;\mathrm{wrost}\;\mathrm{by}\;\mathrm{Equation}\;(11)\\ 30.\;\;\;\;\;\;\;\;\mathrm{end}\\ 31.\;\;\;\;\;\;\;\mathrm{end}\\ 32.\;\;\;\;\;\;\mathrm{end}\\ 33.\;\;\;\;\;\mathrm{end}\\ 34.\;\;\;\;\;\mathrm{Calculate}\;\mathrm{the}\;\mathrm{objective}\;\mathrm{function}\;\mathrm{fitness}\;\mathrm{value}\;\mathrm{by}\;\mathrm{Xnewm}\;\mathrm{and}\;\mathrm{update}\;\mathrm{Xm}\\ 35.\;\;\;\;\;\mathrm{Calculate}\;\mathrm{the}\;\mathrm{objective}\;\mathrm{function}\;\mathrm{fitness}\;\mathrm{value}\;\mathrm{by}\;\mathrm{Xnewf}\;\mathrm{and}\;\mathrm{update}\;\mathrm{Xf}\;\\ 36.\;\;\;\;\;\mathrm{Update}\;\mathrm{the}\;\mathrm{optimal}\;\mathrm{objective}\;\mathrm{function}\;\mathrm{fitness}\;\mathrm{value}\;\mathrm{and}\;\mathrm{optimal}\;\mathrm{male}\;\mathrm{snake}\;\mathrm{vector}\;\\ 37.\;\;\;\;\;\mathrm{for}\;\mathrm{the}\;\mathrm{male}\;\mathrm{snake}\;\mathrm{population}\;\\ 38.\;\;\;\;\;\mathrm{Update}\;\mathrm{the}\;\mathrm{optimal}\;\mathrm{objective}\;\mathrm{function}\;\mathrm{fitness}\;\mathrm{value}\;\mathrm{and}\;\mathrm{optimal}\;\mathrm{female}\;\mathrm{snake}\;\mathrm{vector}\;\\ 39.\;\;\;\;\;\mathrm{of}\;\mathrm{the}\;\mathrm{female}\;\mathrm{snake}\;\mathrm{population}\;\\ 40.\;\;\;\;\;\mathrm{Update}\;\mathrm{the}\;\mathrm{global}\;\mathrm{optimal}\;\mathrm{objective}\;\mathrm{function}\;\mathrm{fitness}\;\mathrm{value}\;\mathrm{gbest}\text{\_}t\\ 41.\;\;\;\;\;\mathrm{and}\;\mathrm{global}\;\mathrm{optimal}\;\mathrm{snake}\;\mathrm{vector}\;\mathrm{Xfood}\;\\ 42.\;\mathrm{end}\; \end{array}$


### 2.4. Hierarchical Task Planning Method

Based on the level of completeness of the initial target information obtained by the platform, the AUV task planning can be typically divided into two categories: one is task planning under conditions of initially complete target information, and the other is task planning under conditions of initially incomplete target information.

#### 2.4.1. Task Planning Under Initial Complete Goal Information

**Figure 4:**
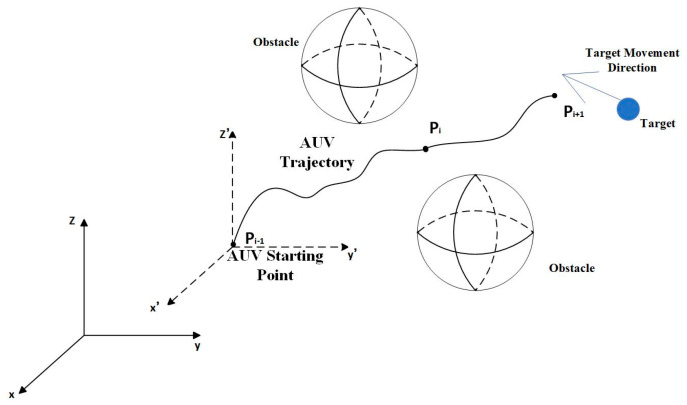
Schematic diagram of AUV planning under complete information conditions.

The schematic diagram of AUV planning under the condition of initially complete target information is shown in Figure 4. It indicates that the AUV has obtained relatively complete motion information about the target during the planning phase.

**Figure 5:**
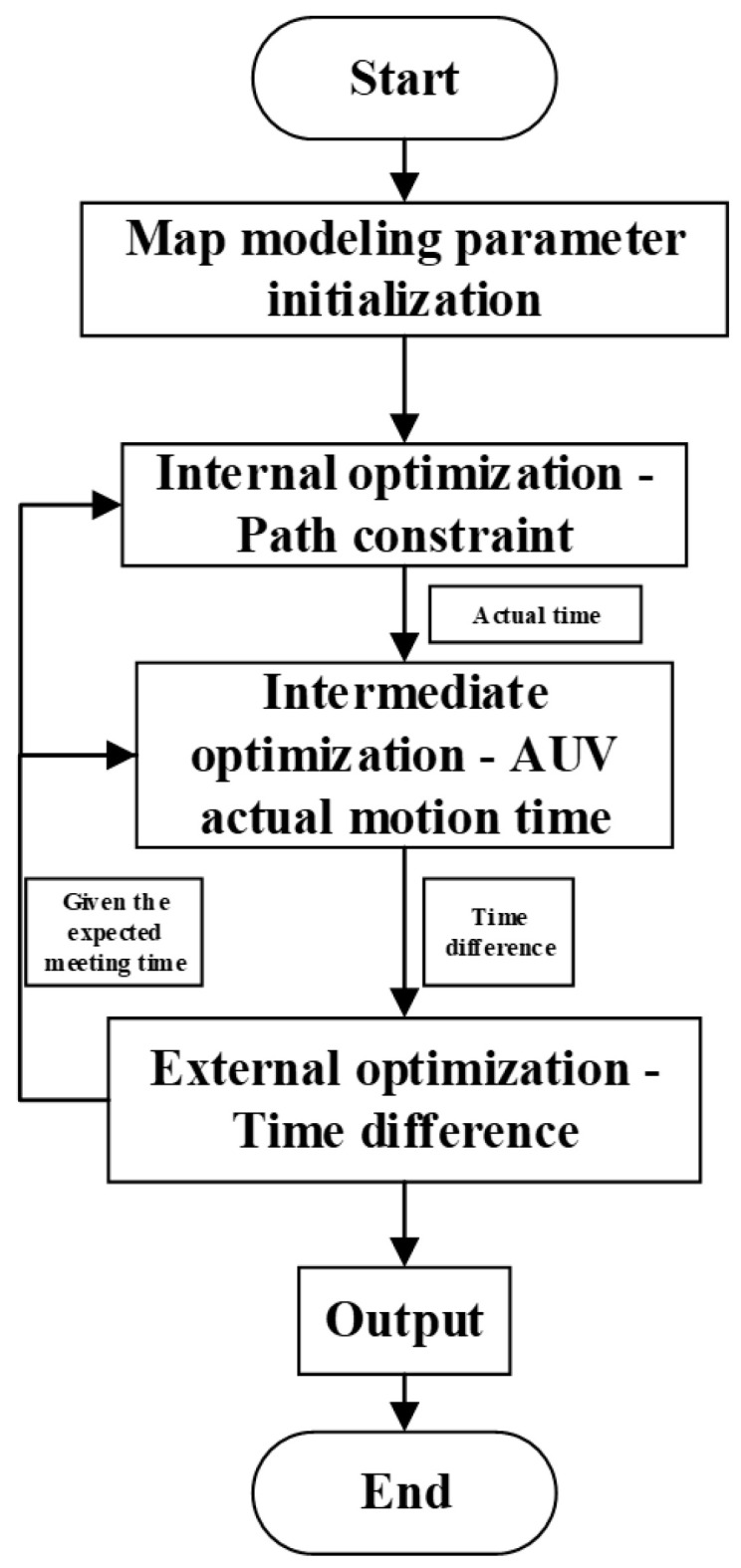
Overall idea diagram of three-layer task planning based on SO algorithm(Complete target information).

The overall design approach for task planning under complete information is shown in Figure 5. In this paper, a three-level optimized task planning based on the snake optimization algorithm is proposed. Given the target location, direction of motion, and speed, the task planning for the AUV aims to minimize the time taken for the AUV to encounter the target. The entire planning process is divided into three levels: external planning, intermediate planning, and internal planning.

External planning focuses on optimizing the difference between the actual movement time of the AUV and the estimated time of encounter between the AUV and the target, which involves optimizing the overall objective function. Intermediate planning optimizes the actual movement time of the AUV. Internal planning optimizes the constraints associated with the AUV’s path planning. By iteratively optimizing the AUV’s actual movement time, intermediate planning aims to achieve the shortest possible time. Meanwhile, it calculates the time difference between the actual movement time and the given estimated time of encounter and feeds this information back to external planning for further optimization.The entire process iterates until an optimal result is achieved.

The objective of the three-level task planning based on the snake optimization algorithm is to minimize the time taken for the AUV to encounter the target, which means the difference between the actual movement time of the AUV and the estimated time of encounter should be as small as possible.Therefore, the objective optimization function of external planning is defined as:

(12) $$\Delta t=\left|t_{auv}-t_{target}\right|,$$

where tauv represents the actual movement time of the AUV; target is the given estimatedtime for the encounter between the AUV and the target, which is also considered as the target movement time; and the difference between the actual movement time of the AUV and the estimated time of encounter is represented by Δt.

The objective of intermediate planning is to minimize the actual movement time of the AUV. The target optimization function is simply the practical movement time of the AUV, denoted as practical time.

Internal planning aims to optimize a better path for the AUV, which involves a multi-objective optimization process. The planning objectives include the optimal combination of path length, smoothness, and safety for the AUV.

The evaluation function for path length is the sum of the distances between each path node. The navigation path “Xi” is represented as a list of “n” waypoints that the AUV needs to follow. Each waypoint corresponds to a path node in the search map, with coordinates $P_{ij}=\left(x_{ij},y_{ij},z_{ij}\right)$. By representing the Euclidean distance between two nodes as $∥\vec{P_{ij}P_{i,j+1}}∥$, the cost F1 associated with path length can be calculated as:

(13) $$F_{1}\left(X_{i}\right)=\sum_{j=1}^{n-1}∥\vec{P_{ij}P_{i,j+1}}∥,$$

**Figure 6:**
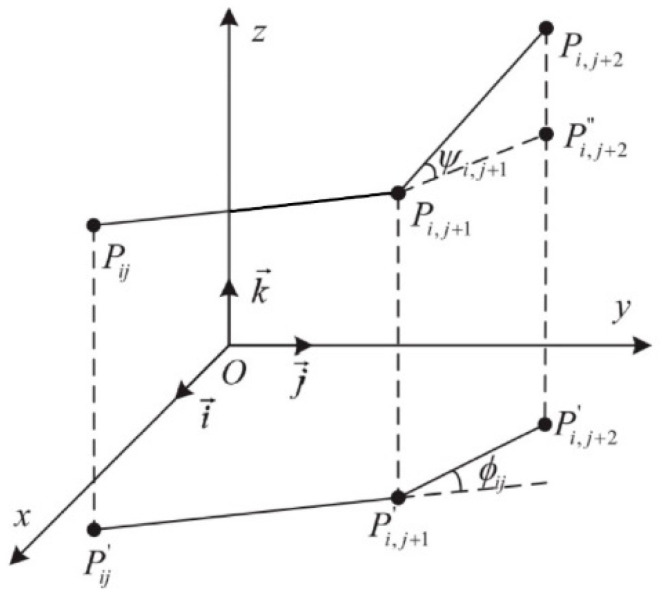
Schematic diagram for calculating turning and climbing angles.

The evaluation function for path smoothness is a function of the path’s turning angles, as shown in Figure 6.

The turning angle *φ_ij_* is the angle between two consecutive path segments and the projections of these segments onto the horizontal *Oxy* plane, represented as vectors. Let $\vec{k}$ be the unit vector in the z-axis direction. The projection vectors can be calculated as:

(14) $$\vec{P_{ij}^{\prime }P_{i,j+1}^{\prime }}=\vec{k}\times \left(\vec{P_{ij}P_{i,j+1}}\times \vec{k}\right),$$

Therefore, the turning angle is calculated as follows:

(15) $$\phi_{ij}=\arctan\left(\frac{∥\vec{P_{ij}^{\prime }P_{i,j+1}^{\prime }}\times \vec{P_{i,j+1}^{\prime }P_{i,j+2}^{\prime }}∥}{\vec{P_{ij}^{\prime }P_{i,j+1}^{\prime }}\cdot \vec{P_{i,j+1}^{\prime }P_{i,j+2}^{\prime }}}\right),$$

The climbing angle *ψ_ij_* is the angle between the path segment $\vec{P_{i,j}P_{i,j+1}}$ and its projection onto the horizontal plane $\vec{P_{ij}^{\prime }P_{i,j+1}^{\prime }}$. It is given by the following formula:

(16) $$\psi_{ij}=\arctan\left(\frac{z_{i,j+1}-z_{ij}}{∥\vec{P_{ij}^{\prime }P_{i,j+1}^{\prime }}∥}\right),$$

The calculation of the smoothing cost is as follows:

(17) $$F_{2}\left(X_{i}\right)=a_{1}\sum_{j=1}^{n-2}\phi_{ij}+a_{2}\sum_{j=1}^{n-1}\left|\psi_{ij}-\psi_{i,j-1}\right|,$$

where *a*_1_ and *a*_2_ are the penalty coefficients for the turning angle and climbing angle, respectively.

**Figure 7:**
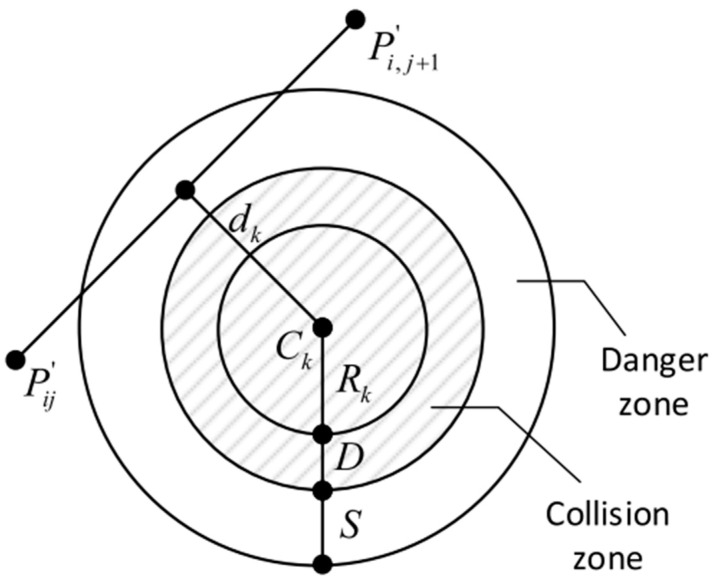
Determination of threat costs.

The planned path must meet safety conditions. Let *K* be the set of all collision zones, assuming that each collision zone has a center coordinate *C_k_* and a radius *R_k_* for its projection, as shown in Figure 7. The cost of a threat increases as the distance from the threat center decreases. For a given path segment $∥\vec{P_{ij}P_{i,j+1}}∥$, the associated threat cost is proportional to its distance *d_k_* from *C_k_*. By considering the safety margin distance *D* of the AUV and the hazardous distance *S* to the collision zone, the threat cost *F_3_* at waypoint *P_ij_* for the obstacle set *K* is calculated as follows:

(18) $$\{\begin{array}{c} F_{3}\left(X_{i}\right)=\sum_{j=1}^{n-1}\sum_{k=1}^{K}T_{k}\left(\vec{P_{ij}P_{i,j+1}}\right),\\ T_{k}\left(\vec{P_{ij}P_{i,j+1}}\right)=\{\begin{array}{cc} 0, & \;\mathrm{if}\;d_{k}>S+D+R_{k}\\ \left(S+D+R_{k}\right)-d_{k}, & \;\mathrm{if}\;D+R_{k}<d_{k}\leq S+D+R_{k}\\ \infty , & \;\mathrm{if}\;d_{k}\leq D+R_{k}. \end{array} \end{array},$$

By considering the optimality, safety, and feasibility constraints associated with the path Xi, the overall cost function can be defined in the following form:

(19) $$F\left(X_{i}\right)=\sum_{k=1}^{3}b_{k}F_{k}\left(X_{i}\right),$$

where *b_k_* is the weighting coefficient, and *F*_1_(*X_i_*) to *F*_3_(*X_i_*) are the costs associated with path length, path smoothness, and safety, respectively. The decision variable is *X_i_*, which includes a list of n waypoints $P_{ij}=\left(x_{ij},y_{ij},z_{ij}\right)$.

The detailed process of the three-level optimization task planning based on the snake optimization algorithm under initial complete information conditions is as follows:

1. Initialize parameters, providing the initial positions, movement speeds, etc. of the AUV and the target;
2. Read map parameters, perform environmental modeling, and set obstacles;
3. Use an external optimization algorithm to estimate the predicted encounter time *t_target_* between the AUV and the *t_target_*;
4. Derive the end position, which is the predicted encounter point, from *t_target_*, and perform task planning for the AUV based on this position;
5. Internal optimization aims to minimize the constraint costs and returns the corresponding actual movement time of the AUV to the intermediate optimizer;
6. The intermediate optimizer optimizes the actual movement time of the AUV to achieve the shortest time *t_auv_*;
7. Calculate the difference between the AUV movement time *t_auv_* provided by the intermediate optimizer and the predicted encounter time *t_target_*. Return this time difference to the external optimizer for further optimization to obtain the minimum time difference and provide the optimal predicted encounter time for both.

**Figure 8:**
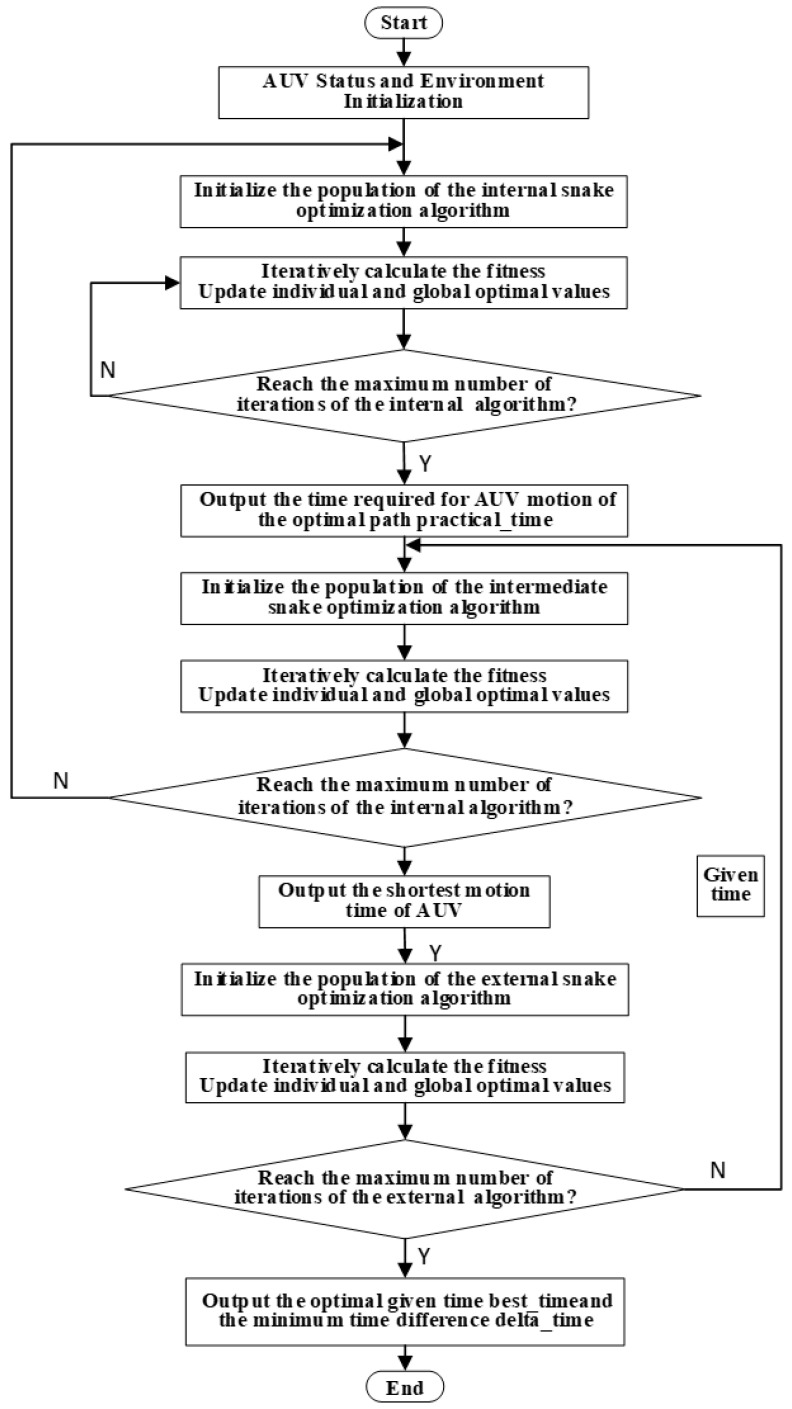
Complete information for the overall task planning flowchart.

The flowchart of the task planning method under complete target information is illustrated in Figure 8. Under the condition of initial complete information, the parameter initialization and environment modeling are performed first. Next, the estimated encounter time is obtained through external optimization, and the end position is obtained according to the estimated encounter time. Then the constraint cost is minimized by internal optimization, and the shortest time of the actual motion is obtained by the intermediate optimizer. Finally, the difference between the two times is calculated, and the difference is returned to the external optimizer for optimization, where the optimal time for the meeting is obtained.

The implementation of the three-level optimization task is based on the snake algorithm framework, and the main difference between the three loops lies in the objective function. The pseudocode of the path planning of the internal loop is shown in Algorithm 2. The path planning of the internal loop serves as the objective function of the external loop, achieving a three-level nested optimization. The target endpoint of the path planning of the internal loop is calculated based on the time design variables and target motion information of the external loop. The objective function of the internal loop is the fitness value calculated using Equation (19) based on the planned path. The objective function of the external loop is to calculate the error between the navigation time corresponding to the optimal path achieved through internal loop optimization under a given time optimization variable and a certain time optimization variable in the external loop. The objective function of the intermediate loop is to calculate the move time of the planning path. The pseudocode of the external and internal loop objectiveness function is in Algorithm 3.
**Algorithm 2.** Internal Optimization Function$\begin{array}{l} \mathrm{Internal}\;\mathrm{optimizer}\;\mathrm{funciton}\text{:}\;\mathrm{So}\text{\_}\mathrm{path}\\ \mathrm{Input}\text{:}\mathrm{pop}\text{\_}\mathrm{path},\mathrm{Iteration}\text{\_}\mathrm{path},\mathrm{fobj}\text{\_}\mathrm{path},\;{\dim\text{\_}\mathrm{path},X}_{\min}{\text{\_}\mathrm{path},X}_{\max}\text{\_}\mathrm{path}\\ \mathrm{Output}\text{:}\mathrm{Xfood},\mathrm{fval},\mathrm{gbest}\text{\_}\mathrm{path},\mathrm{path}\text{\_}\mathrm{time}\\ 1{.\;\mathrm{Initialize}\;\mathrm{ThresholdQ}\;\mathrm{ThresholdT},c}_{1}{,c}_{2}{,c}_{3}\\ 2{.\;X=\mathrm{initializtion}(\mathrm{pop}\text{\_}\mathrm{path},\dim\text{\_}\mathrm{path},X}_{\max}{\text{\_}\mathrm{path},X}_{\min}\text{\_}\mathrm{path})\\ 3.\;\mathrm{Calculate}\;\mathrm{fobj}\text{\_}\mathrm{path}\;\mathrm{function}\;\mathrm{fitness}\;\mathrm{value}\;\mathrm{for}\;\mathrm{each}\;X\text{\_}\mathrm{path}\\ 4.\;[\mathrm{GYbest},\mathrm{gbest}\text{\_}\mathrm{path}]=\min(\mathrm{fitness}),\mathrm{Xfood}=X\text{\_}\mathrm{path}(\mathrm{gbest},\text{:})\\ 5.\;\mathrm{Nm}=\mathrm{round}(\mathrm{pop}/2),\;\mathrm{Nf}=\mathrm{pop}-\mathrm{Nm}\\ 6.\;\mathrm{Xm}=X\text{\_}\mathrm{path}(1\text{:}\mathrm{Nm},\text{:}),\;\mathrm{Xf}=X\text{\_}\mathrm{path}(\mathrm{Nm}+1\text{:}\mathrm{pop},\text{:})\\ 7.\;\mathrm{fitness}\text{\_}m=\mathrm{fitness}(1\text{:}\mathrm{Nm}),\;\mathrm{bestX}\text{\_}\mathrm{path}\text{\_}m=\mathrm{bestX}(1\text{:}\mathrm{Nm},\text{:})\;\%\mathrm{Male}\;\mathrm{snake}\;\mathrm{population}\;\\ 8.\;[\mathrm{fitnessBest}\text{\_}m,\mathrm{gbest}1]=\min(\mathrm{fitness}\text{\_}m),\;\mathrm{Xbest}\text{\_}m=\mathrm{Xm}(\mathrm{gbest}1,\text{:})\\ 9.\;\mathrm{fitness}\text{\_}f=\mathrm{fitness}(\mathrm{Nm}+1\text{:}\mathrm{pop}),\;\mathrm{bestX}\text{\_}\mathrm{path}\text{\_}f=\mathrm{bestX}(\mathrm{Nm}+1\text{:}\mathrm{pop},\text{:})\;\%\mathrm{Female}\;\mathrm{snake}\;\mathrm{population}\\ 10.\;[\mathrm{fitnessBest}\text{\_}f,\mathrm{gbest}2]=\min(\mathrm{fitness}\text{\_}f),\;\mathrm{Xbest}\text{\_}f=\mathrm{Xm}(\mathrm{gbest}2,\text{:})\\ 11.\;\mathrm{for}\;i=1\text{:}\mathrm{Iteration}\\ 12{.\;\;\mathrm{Temp}=\exp(-((t)/\mathrm{Iteration})),\;Q=c}_{1}\times \exp(((t-\mathrm{Iteration})/\mathrm{Iteration}))\\ 13.\;\;\;\mathrm{if}\;Q<\mathrm{Threshold}\\ 14.\;\;\;\;\;\mathrm{update}\;\mathrm{Xnewm}\;\mathrm{use}\;\mathrm{Xm}\;\mathrm{by}\;\mathrm{Equation}\;(5)\\ 15.\;\;\;\;\;\mathrm{update}\;\mathrm{Xnewf}\;\mathrm{use}\;\mathrm{Xf}\;\mathrm{by}\;\mathrm{Equation}\;(5)\;\\ 16.\;\;\;\mathrm{else}\\ 17.\;\;\;\;\mathrm{if}\;\mathrm{Temp}>\mathrm{Thresold}2\\ 18.\;\;\;\;\;\mathrm{update}\;\mathrm{Xnewm}\;\mathrm{use}\;\mathrm{Xbest}\;\mathrm{by}\;\mathrm{Equation}\;(7)\\ 19.\;\;\;\;\;\mathrm{update}\;\mathrm{Xnewf}\;\mathrm{use}\;\mathrm{Xbest}\;\mathrm{by}\;\mathrm{Equation}\;(7)\;\\ 20.\;\;\;\;\mathrm{else}\\ 21.\;\;\;\;\;\mathrm{if}\;\mathrm{rand}>0.6\\ 22.\;\;\;\;\;\;\mathrm{update}\;\mathrm{Xnewm}\;\mathrm{use}\;\mathrm{Xm}\;\mathrm{by}\;\mathrm{Equation}(8)\\ 23.\;\;\;\;\;\;\mathrm{update}\;\mathrm{Xnewf}\;\mathrm{use}\;\mathrm{Xf}\;\mathrm{by}\;\mathrm{Equation}\;(8)\\ 24.\;\;\;\;\;\mathrm{else}\\ 25.\;\;\;\;\;\;\mathrm{update}\;\mathrm{Xnewm}\;\mathrm{use}\;\mathrm{Xm}\;\mathrm{by}\;\mathrm{Equation}\;(8)\;\mathrm{replace}\;\mathrm{Fm}\;\mathrm{with}\;\mathrm{Mm}\;\\ 26.\;\;\;\;\;\;\mathrm{update}\;\mathrm{Xnewf}\;\mathrm{use}\;\mathrm{Xf}\;\mathrm{by}\;\mathrm{Equation}\;(8)\;\mathrm{replace}\;\mathrm{Fm}\;\mathrm{with}\;\mathrm{Mm}\;\\ 27.\;\;\;\;\;\;\;\mathrm{if}\;\mathrm{rand}>0.5\\ 28.\;\;\;\;\;\;\;\mathrm{update}\;\mathrm{Xnewm}\;\mathrm{wrost}\;\mathrm{by}\;\mathrm{Equation}\;(11)\\ 29.\;\;\;\;\;\;\;\mathrm{update}\;\mathrm{Xnewf}\;\mathrm{wrost}\;\mathrm{by}\;\mathrm{Equation}\;(11)\\ 30.\;\;\;\;\;\;\;\mathrm{end}\\ 31.\;\;\;\;\;\;\mathrm{end}\\ 32.\;\;\;\;\;\mathrm{end}\\ 33.\;\;\;\;\mathrm{end}\\ 34.\;\;\;\;\mathrm{Calculate}\;\mathrm{the}\;\mathrm{objective}\;\mathrm{function}\;\mathrm{fitness}\;\mathrm{value}\;\mathrm{by}\;\mathrm{Xnewm}\;\mathrm{and}\;\mathrm{update}\;\mathrm{Xm}\\ 35.\;\;\;\;\mathrm{Calculate}\;\mathrm{the}\;\mathrm{objective}\;\mathrm{function}\;\mathrm{fitness}\;\mathrm{value}\;\mathrm{by}\;\mathrm{Xnewf}\;\mathrm{and}\;\mathrm{update}\;\mathrm{Xf}\;\\ 36.\;\;\;\;\mathrm{Update}\;\mathrm{the}\;\mathrm{optimal}\;\mathrm{objective}\;\mathrm{function}\;\mathrm{fitness}\;\mathrm{value}\;\mathrm{and}\;\mathrm{optimal}\;\mathrm{male}\;\mathrm{snake}\;\mathrm{vector}\;\\ 37.\;\;\;\;\mathrm{for}\;\mathrm{the}\;\mathrm{male}\;\mathrm{snake}\;\mathrm{population}\;\\ 38.\;\;\;\;\mathrm{Update}\;\mathrm{the}\;\mathrm{optimal}\;\mathrm{objective}\;\mathrm{function}\;\mathrm{fitness}\;\mathrm{value}\;\mathrm{and}\;\mathrm{optimal}\;\mathrm{female}\;\mathrm{snake}\;\mathrm{vector}\;\\ 39.\;\;\;\;\mathrm{of}\;\mathrm{the}\;\mathrm{female}\;\mathrm{snake}\;\mathrm{population}\;\\ 40.\;\;\;\;\mathrm{Update}\;\mathrm{the}\;\mathrm{global}\;\mathrm{optimal}\;\mathrm{objective}\;\mathrm{function}\;\mathrm{fitness}\;\mathrm{value}\;\mathrm{gbest}\text{\_}t\\ 41.\;\;\;\;\mathrm{and}\;\mathrm{global}\;\mathrm{optimal}\;\mathrm{snake}\;\mathrm{vector}\;\mathrm{Xfood}\;\\ 42.\;\mathrm{end}\; \end{array}$

**Algorithm 3.** Objective Function of Intial Complete Target Information ConditionsInternal objective Function

$\begin{array}{l} \mathrm{Internal}\;\mathrm{optimizer}\;\mathrm{object}\;\mathrm{funtion}\text{:}\;\mathrm{MyCost}\text{\_}\mathrm{path}\\ \mathrm{Input}\text{:}X\text{\_}\mathrm{path},\mathrm{UUV},\mathrm{UUV}\text{\_}v\\ \mathrm{Output}\text{:}\cos t,\mathrm{path}\text{\_}\mathrm{time}\\ 1{.\;P}_{\mathrm{Terminal}}=\mathrm{UUV}+\mathrm{path}\text{\_}\mathrm{time}\times \mathrm{UUV}\text{\_}v\\ 2{.\;\mathrm{Path}=[X\text{\_}\mathrm{path};P}_{\mathrm{Terminal}}]\\ 3{.\;\mathrm{Calculate}\;\mathrm{path}\;\mathrm{length}\;\cos t\;F}_{1}\;\mathrm{use}\;\mathrm{Path}\;\mathrm{by}\;\mathrm{Equation}\;(7)\\ 4{.\;\mathrm{Calculate}\;\mathrm{smoothing}\;\cos t\;F}_{2}\;\mathrm{use}\;\mathrm{Path}\;\mathrm{by}\;\mathrm{Equation}\;(17)\;\\ 5{.\;\mathrm{Calculate}\;\mathrm{threat}\;\cos t\;F}_{3}\;\mathrm{use}\;\mathrm{Path}\;\mathrm{by}\;\mathrm{eq}(\mathrm{Equation}\;)\\ 6.\;\mathrm{Calculate}\;\mathrm{fitness}\;\mathrm{value}\;\cos t\;\mathrm{by}\;\mathrm{Equation}\;(19)\\ 7.\;\mathrm{Calculate}\;\mathrm{path}\text{\_}\mathrm{time}\;\mathrm{by}\;\mathrm{Path} \end{array}$

Intermediate objective Function

$\begin{array}{l} \mathrm{external}\;\mathrm{optimizer}\;\mathrm{object}\;\mathrm{funtion}\text{:}\;\mathrm{MyCost}\text{\_}\mathrm{time}\\ \mathrm{Input}\text{:}X\text{\_}\mathrm{time},\mathrm{UUV}\\ \mathrm{Output}\text{:}\mathrm{best}\text{\_}\mathrm{time},\mathrm{bestX}\\ 1.\;\mathrm{fobj}\text{\_}\mathrm{path}=\text{@}\mathrm{MyCost}\text{\_}\mathrm{path}\\ 2.\;[\mathrm{bestX}\text{\_}\mathrm{path},\mathrm{path}\text{\_}\mathrm{time}]=\mathrm{So}\text{\_}\mathrm{path}(\mathrm{pop}\text{\_}\mathrm{path},\mathrm{Iteration}\text{\_}\mathrm{path},\mathrm{fobj}\text{\_}\mathrm{path},\dim\text{\_}\mathrm{path},\\ 3.\;X\text{\_}\max,X\text{\_}\min,X\text{\_}\mathrm{time},\mathrm{UUV})\\ 4.\;\mathrm{best}\text{\_}\mathrm{time}=\mathrm{path}\text{\_}\mathrm{time} \end{array}$

external objective Function

$\begin{array}{l} \mathrm{external}\;\mathrm{optimizer}\;\mathrm{object}\;\mathrm{funtion}\text{:}\;\mathrm{MyCost}\text{\_}\mathrm{deltatime}\\ \mathrm{Input}\text{:}X\text{\_}\mathrm{time},\mathrm{UUV}\\ \mathrm{Output}\text{:}\mathrm{delta}\text{\_}\mathrm{time},\mathrm{bestX},\mathrm{path}\text{\_}\mathrm{time}\\ \mathrm{fobj}\text{\_}\mathrm{delta}=\text{@}\mathrm{MyCost}\text{\_}\mathrm{delta}\\ {}[\mathrm{bestX},\mathrm{path}\text{\_}\mathrm{time}]=\mathrm{So}\text{\_}\mathrm{time}(\mathrm{pop}\text{\_}\mathrm{time},\mathrm{Iteration}\text{\_}\mathrm{time},\mathrm{fobj}\text{\_}\mathrm{time},\dim\text{\_}\mathrm{time},\\ X\text{\_}\mathrm{time}\text{\_}\max,X\text{\_}\mathrm{time}\text{\_}\min,X\text{\_}\mathrm{time},\mathrm{UUV})\\ \mathrm{delta}\text{\_}\mathrm{time}=X\text{\_}\mathrm{time}-\mathrm{path}\text{\_}\mathrm{time} \end{array}$



#### 2.4.2. Task Planning Under Conditions of Initially Incomplete Target Information

**Figure 9:**
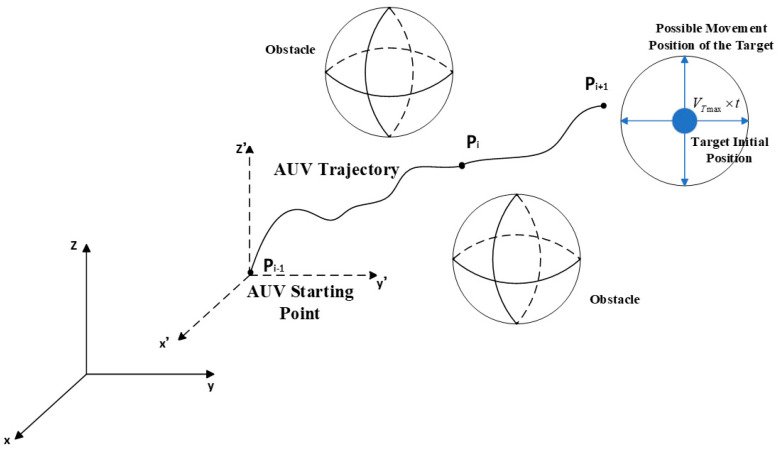
Schematic diagram of AUV planning under incomplete information conditions.

The schematic diagram of AUV planning under the condition of initially incomplete target information is shown in Figure 9.

In this scenario, the AUV only knows the approximate initial location of the target, while the specific direction of the target’s motion remains unknown. However, based on practical engineering experience, an estimation of the maximum speed of the target can be made. When the AUV is deployed from the platform, this moment is considered as time zero. At any given time t, the target’s location will be somewhere within a circle centered at the initial point with a radius equal to the maximum distance the target could have traveled at its maximum speed since time zero.

**Figure 10:**
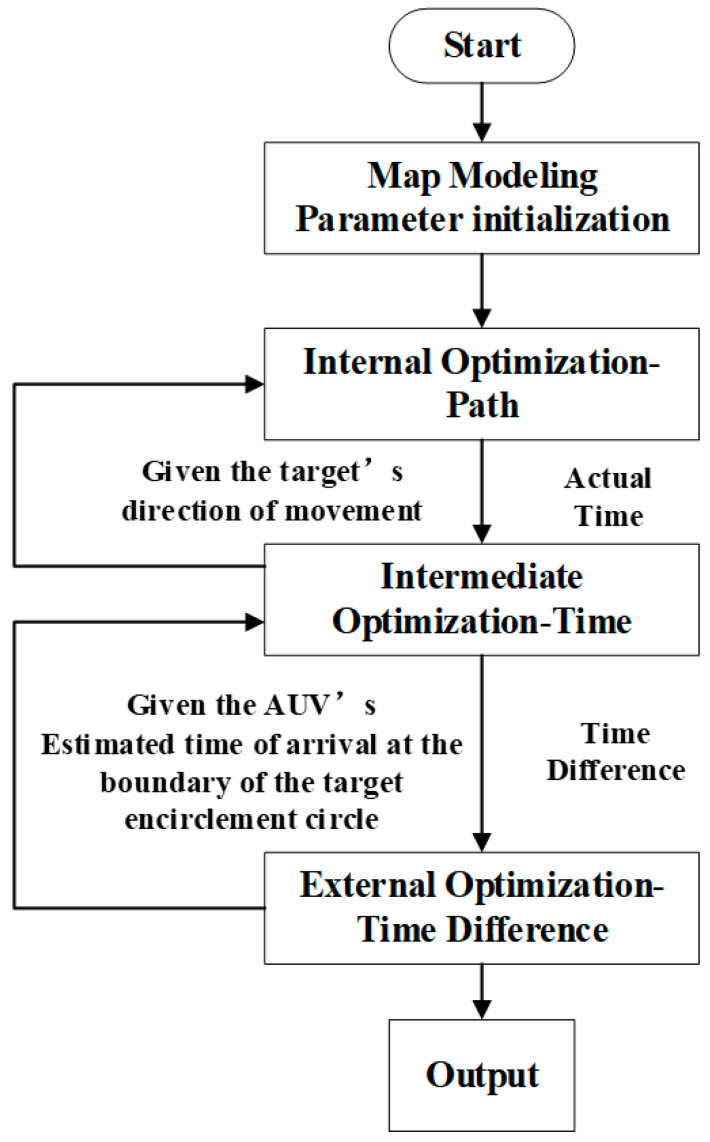
Overall idea diagram of three-layer task planning based on SO algorithm (incomplete target information).

The overall design approach for task planning under incomplete information is shown in Figure 10. This paper proposes a three-level optimization task planning based on the snake optimization algorithm. Given the initial position information of the target and its maximum speed, the task planning for the AUV is carried out. In this case, the target’s movement range can be approximated as a circular area. Assuming the maximum speed of the target is *V_max_*, the movement time is t1, and the azimuth angle is *θ*, this information can determine the boundary of the circle. The ultimate goal is to make the AUV reach this circle in the shortest time while minimizing the size of the encircled area. The entire planning process is still divided into three levels: external planning, intermediate planning, and internal planning.

External planning mainly optimizes the difference between the actual time it takes for the AUV to reach the boundary of the encircled area and the estimated time it is expected to arrive, which involves optimizing the overall objective function. Intermediate planning focuses on finding the minimum encircled area or the shortest actual movement time for the AUV given different movement azimuth angles *θ* of the target. Internal planning optimizes the constraints involved in AUV path planning. By continuously providing different *θ* values, intermediate planning aims to find the *θ* that results in the shortest actual movement time for the AUV or the smallest encircled area. The optimized shortest actual movement time obtained from intermediate planning is subtracted from the estimated time provided by external planning to calculate the time difference, which is then returned to external planning for further optimization. The entire process iterates until an optimal result is achieved.

Task planning under incomplete information aims to achieve the goal of the AUV reaching the smallest encircled area of the target in the shortest possible time. Therefore, the objective optimization function ofexternal planningis taken as:

(20) $$\Delta t=\left|t_{auv}-t_{\mathrm{give}}\right|,$$

where $t_{auv}$ represents the actual movement time of the AUV; $t_{\mathrm{give}}$ represents the estimated time for the AUV to reach the boundary of the encircled area; and Δ*t* represents the difference between the actual movement time of the AUV and the estimated encounter time. The objective of this optimization function is to minimize the time difference Δ*t*, which means making the actual movement time of the AUV as close as possible to the given estimated arrival time.

The purpose of intermediate planning is to find the direction angle at which the target moves, resulting in the smallest encircled area, which corresponds to the minimum time for the AUV to actually reach the encircled area.

Due to the unknown direction of the target’s motion, the maximum dispersion circle of the target can be determined based on the given task time and maximum motion speed. If the expected angle of the AUV on the dispersion circle is determined, the endpoint of the path planning can be determined by Equations (21) and (22).

(21) $$X_{end}=X_{ini,tar}+t_{give}*V_{max}*sin\theta ,$$

(22) $$Y_{end}=Y_{ini,tar}+t_{give}*V_{max}*cos\theta ,$$

where $X_{ini,tar}$, $Y_{ini,tar}$ are the coordinates of the initial position of the target, $V_{max}$ is the maximum velocity of the taret, $t_{give}$ is the expected task completion time issued by the outer loop, and *θ* is the design variable of the middle loop

The internal objective optimization function is the shortest actual movement time of the AUV corresponding to the target dispersion circle angle *θ*. The optimal path and time to reach the endpoint of the dispersion circle can be obtained through the inner planning loop. The intermediate loop obtains the shortest navigation time and arrival angle for the AUV to meet the target dispersion circle through angle planning. The external loop optimizes the time to complete the task. The outer loop optimizes the expected task time to minimize the error between the expected task time $t_{give}$ and the actual completion time $t_{auv}$.

The objective function of internal planning is the same as the internal optimization objective function under the complete information scenario.

The detailed process of the three-level optimization task planning based on the snake optimization algorithm under incomplete information conditions is as follows:

1. Initialize parameters: provide the initial positions, movement speeds, etc. of the AUV andthe target.
2. Read map parameters: perform environmental modeling and set obstacles.
3. The external optimizer continuously provides the estimated encounter time (tgive), between the AUV and the target.
4. The intermediate optimizer continuously provides the target dispersion circle angle, θ.
5. Based on the given $t_{\mathrm{give}}$ and *θ*, task planning can be performed for the AUV.
6. Internal optimization minimizes the constraint cost and returns the corresponding actual movement time of the AUV to the intermediate optimizer.
7. By continuously adjusting the target movement azimuth angle, the intermediate optimizer finds the shortest time, $t_{auv}$, required for the AUV to actually move and reach the target’s dispersion circle area.
8. The external optimizer calculates the difference between the actual movement time, $t_{auv}$, obtained from the intermediate optimization and the given time, $t_{\mathrm{give}}$. Using the snake optimization algorithm, iterations are performed to continuously adjust the given time, aiming to minimize the absolute value of the difference between the actual movement time required for the AUV to reach the target’s encircled area and the given time. This ensures that the AUV can reach the boundary of the smallest encircled area of the target in the shortest possible time.

**Figure 11:**
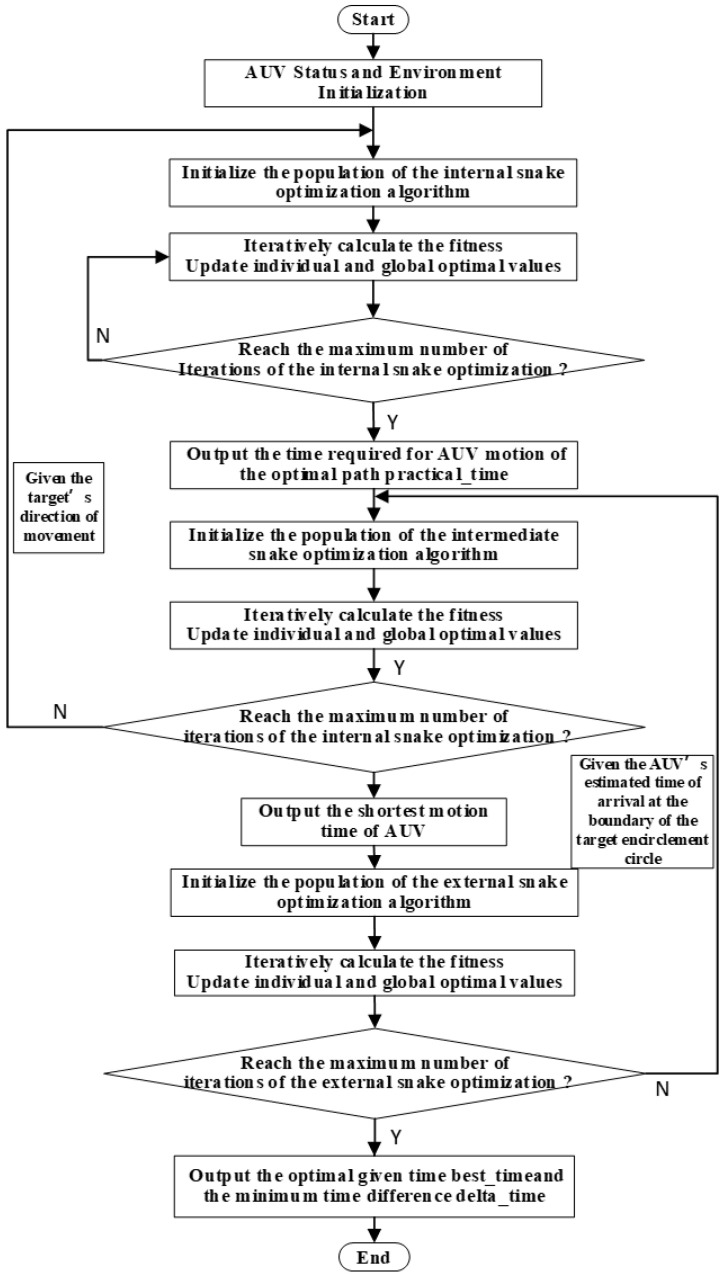
Incomplete information overall task planning flowchart.

The detailed task planning process is shown in Figure 11. The mission planning process under the condition of initial incomplete target information is generally similar to that under complete target information, except that the estimated encounter time obtained by the external optimization algorithm becomes the estimated time for the AUV to reach the encirclement boundary.

The program structure of each of the three layers is basically similar (Algorithm 2). The main difference between the three layers lies in the objective function. The external layer remains the same as the complete condition, with the optimization task completion time error. The intermediate layer optimization objective function is to findthe shortest navigation time path toreach the target dispersion circle andcorresponding angle design variable *θ*, the path achieved through nested internal layer path optimization programs. The objective function of the internal layer path planning remains unchanged when compared to the completeness condition. The pseudocode of the external and internal loop objectiveness function is in Algorithm 4.
**Algorithm 4.** Objective Function of Incomplete Intial Target Information ConditionsInternal objective Function$\begin{array}{l} \mathrm{Internal}\;\mathrm{optimizer}\;\mathrm{object}\;\mathrm{funtion}\text{:}\;\mathrm{MyCost}\text{\_}\mathrm{path}\\ \mathrm{Input}\text{:}X\text{\_}\mathrm{path},\mathrm{UUV},\mathrm{UUV}\text{\_}v\\ \mathrm{Output}\text{:}\cos t,\mathrm{path}\text{\_}\mathrm{time}\\ 1{.\;P}_{\mathrm{Terminal}}=\mathrm{UUV}+\mathrm{path}\text{\_}\mathrm{time}\times \mathrm{UUV}\text{\_}v\\ 2{.\;\mathrm{Path}=[X\text{\_}\mathrm{path};P}_{\mathrm{Terminal}}]\\ 3{.\;\mathrm{Calculate}\;\mathrm{path}\;\mathrm{length}\;\cos t\;F}_{1}\;\mathrm{use}\;\mathrm{Path}\;\mathrm{by}\;\mathrm{Equation}\;(7)\\ 4{.\;\mathrm{Calculate}\;\mathrm{smoothing}\;\cos t\;F}_{2}\;\mathrm{use}\;\mathrm{Path}\;\mathrm{by}\;\mathrm{Equation}\;(17)\;\\ 5{.\;\mathrm{Calculate}\;\mathrm{threat}\;\cos t\;F}_{3}\;\mathrm{use}\;\mathrm{Path}\;\mathrm{by}\;\mathrm{Equation}\;(18)\\ 6.\;\mathrm{Calculate}\;\mathrm{fitness}\;\mathrm{value}\;\cos t\;\mathrm{by}\;\mathrm{Equation}\;(19)\\ 7.\;\mathrm{Calculate}\;\mathrm{path}\text{\_}\mathrm{time}\;\mathrm{of}\;\mathrm{Path} \end{array}$Intermediate objective Function$\begin{array}{l} \mathrm{external}\;\mathrm{optimizer}\;\mathrm{object}\;\mathrm{funtion}\text{:}\;\mathrm{MyCost}\text{\_}\mathrm{theta}\\ {\mathrm{Input}\text{:}X\text{\_}\mathrm{theta},\mathrm{UUV},V}_{\max}{,t}_{\mathrm{give}}\\ \mathrm{Output}\text{:}\mathrm{bestX},\mathrm{path}\text{\_}\mathrm{time}\\ 1{.\;\mathrm{Calculate}\;P}_{\mathrm{end},\mathrm{tar}}{\;\mathrm{use}\;\mathrm{UUV},V}_{\max}{,t}_{\mathrm{give}}\;X\text{\_}\mathrm{theta},\mathrm{by}\;\mathrm{Equation}\;(20),\mathrm{Equation}\;(21)\\ 2.\;\mathrm{fobj}\text{\_}\mathrm{theta}=\text{@}\mathrm{MyCost}\text{\_}\mathrm{path}\\ 3.\;[\mathrm{bestX},\mathrm{path}\text{\_}\mathrm{time}]=\mathrm{So}\text{\_}\mathrm{path}(\mathrm{pop}\text{\_}\mathrm{path},\mathrm{Iteration}\text{\_}\mathrm{path},\mathrm{fobj}\text{\_}\mathrm{path},\dim\text{\_}\mathrm{path},\\ 4{.\;X\text{\_}\mathrm{path}\text{\_}\max,X\text{\_}\mathrm{theta}\text{\_}\min,P}_{\mathrm{end},\mathrm{tar}}) \end{array}$External objective Function$\begin{array}{l} \mathrm{external}\;\mathrm{optimizer}\;\mathrm{object}\;\mathrm{funtion}\text{:}\;\mathrm{MyCost}\text{\_}\mathrm{deltatime}\\ \mathrm{Input}\text{:}X\text{\_}\mathrm{deltatime},\mathrm{UUV}\\ \mathrm{Output}\text{:}\mathrm{delta}\text{\_}\mathrm{deltatime},\mathrm{bestX},\mathrm{bestX}\text{\_}\mathrm{theta},\mathrm{path}\text{\_}\mathrm{time}\\ 1.\;\mathrm{fobj}\text{\_}\mathrm{theta}=\text{@}\mathrm{MyCost}\text{\_}\mathrm{theta}\\ 2.\;[\mathrm{bestX}\text{\_}\mathrm{theta},\mathrm{bestX},\mathrm{path}\text{\_}\mathrm{time}]=\mathrm{So}\text{\_}\mathrm{theta}(\mathrm{pop}\text{\_}\mathrm{theta},\mathrm{Iteration}\text{\_}\mathrm{theta},\mathrm{fobj}\text{\_}\mathrm{theta},\dim\text{\_}\mathrm{theta},\\ 3.\;X\text{\_}\mathrm{theta}\text{\_}\max,X\text{\_}\mathrm{theta}\text{\_}\min,X\text{\_}\mathrm{time},X\text{\_}\mathrm{deltatime},\mathrm{UUV})\\ 4.\;\mathrm{delta}\text{\_}\mathrm{time}=X\text{\_}\mathrm{time}-\mathrm{path}\text{\_}\mathrm{time} \end{array}$

## 3. Simulation Results and Analysis

To verify the effectiveness of the proposed mission planning method, simulation experiments were conducted on an Intel i9-13900H with 32 GB of RAM (Intel, Santa Clara, CA, USA) using MATLAB R2018b. We designed simulation experiments for both complete and incomplete target information conditions separately.

### 3.1. Mission Planning Under Initial Complete Target Information Conditions

The input parameters for mission planning under complete information are shown in Table 3.

The initialization position and speed of AUV and target are determined by map scene size, AUV, and target motion capability. Initial Given Time can be estimated by the relative distance between the initial AUV and the target, and the algorithm will automatically iterate without affecting the final result. Path node count determines the speed and smoothness of path planning. If it is too small, it will lead to obvious path mutations, which are not conducive to obstacle avoidance and result in no solution. If it is too large, it will lead to a significant increase in planning time. Due to nested planning, it will present a geometric increase in planning time, generally ranging from 10 to 20.

The snake optimization algorithm population size and iteration count directly affect the accuracy and speed of planning; increasing them can improve the accuracy of solving the problem, facilitate escaping from local optimal solutions, reduce the speed of accelerating planning, and reduce planning time, but theywill affect the accuracy of solving. When selecting, it is necessary to repeatedly try and match based on the actual situation. If the solution accuracy meets the requirements, the numerical value can be minimized to improve efficiency. If the solution accuracy does not meet the requirements or the optimal solution cannot be found, the numerical value should be increased. The range of food judgment value and temperature judgment value is [0, 1], which affects the frequency of two behaviors;the typical value of food judgment value is 0.25, and the typical value of temperature judgment value is 0.6.

Listed in the Table 3, and the detailed parameters of initialization, internal optimization algorithm, intermediate optimization algorithm, and external optimization algorithm are given. The AUV mission under complete information conditions was planned, and the software was run 10 times to obtain the simulation results shown in Table 4.

**Figure 12:**
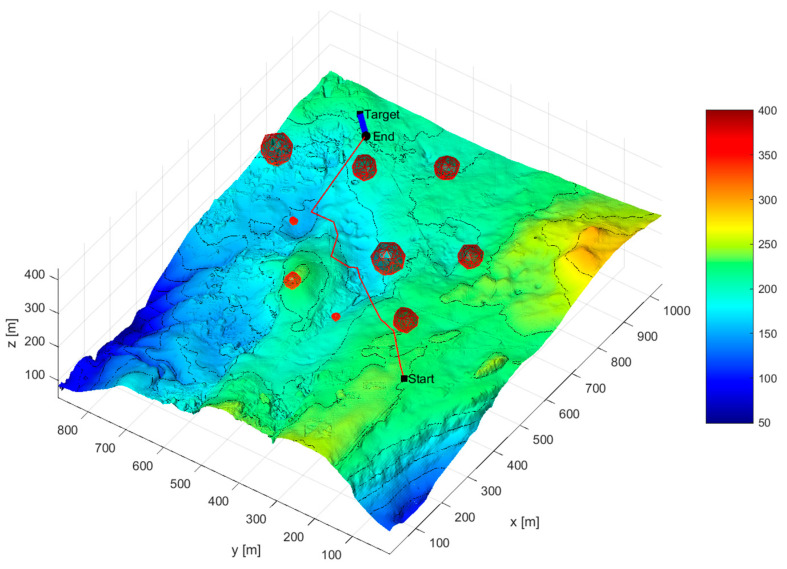
Complete information scenario overall task planning diagram (three-dimensional diagram).

**Figure 13:**
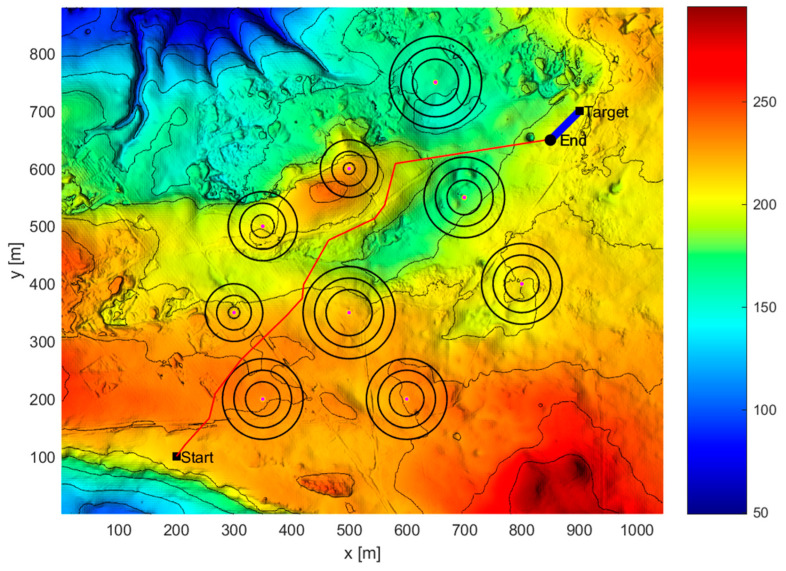
Complete information scenario overall task planning diagram (vertical view).

Figure 12 and Figure 13 show the simulation diagram of AUV mission planning. The red curve represents the AUV path, and the yellow curve represents the target path. “Start” is the initial position of the AUV, “Target” is the initial position of the target, and “End” is the encounter point between the two, which is also the final destination of the target.

As can be seen from Table 4, under complete information conditions, the hierarchical mission planning algorithm can plan an AUV search mission path that satisfies the time threshold requirement for the deviation between the actual mission completion time and the expected mission completion time. The maximum mission planning time deviation is 3.453 s, the minimum mission planning time deviation is 0.101 s, and the average time deviation is 1.41 s. In terms of time deviation rate, the maximum time deviation rate is 3.37%, the minimum time deviation rate is 0.1%, and the average time deviation rate is 1.4%. Figure 12 and Figure 13 show that under complete information conditions, the proposed mission planning method can plan a path that meets multiple constraints, avoids threat areas, and encounters the target as early as possible.

### 3.2. Mission Planning Under Initial Incomplete Target Information Conditions

The input parameters for mission planning under initial incomplete information are shown in Table 5. The input parameters of task planning under the incomplete information scenario are listed in the table, and the detailed parameters of initialization, internal optimization algorithm, intermediate optimization algorithm, and external optimization algorithm are given.

Mission planning was conducted for AUVs under incomplete information conditions, and the software was run 10 times to obtain the simulation results shown in Table 6.

As can be seen from Table 6, under incomplete information conditions, the hierarchical mission planning algorithm can plan an AUV search mission path that satisfies the time threshold requirement for the deviation between the actual mission completion time and the expected mission completion time, while also providing the corresponding direction towards the target. The maximum mission planning time deviation is 3.84 s, the minimum mission planning time deviation is 0.09 s, and the average time deviation is 1.58 s. In terms of time deviation rate, the maximum time deviation rate is 3.6%, the minimum time deviation rate is 0.09%, and the average time deviation rate is 1.52%.

**Figure 14:**
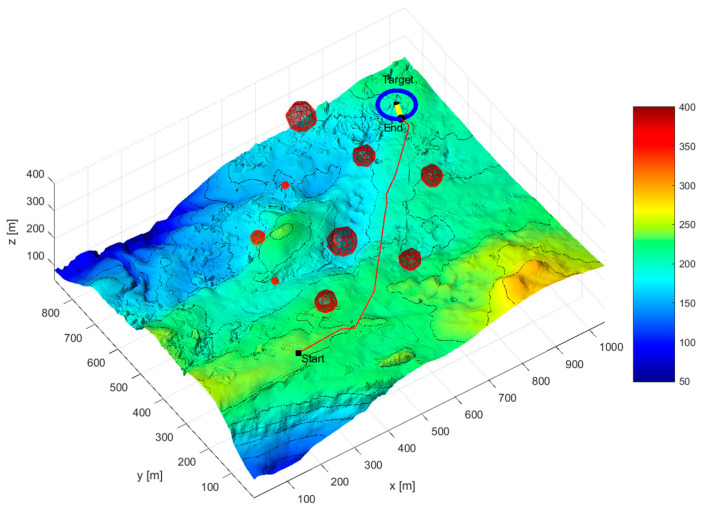
Incomplete information scenario overall task planning diagram (three-dimensional diagram).

**Figure 15:**
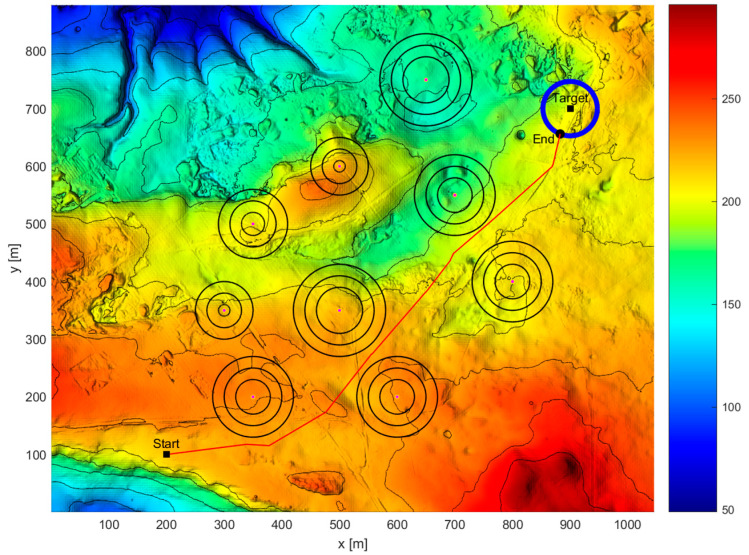
Incomplete information scenario overall task planning diagram (vertical view).

The task planning results are shown in Figure 14 and Figure 15. The red curve is the AUV path, and the yellow curve is the target path. “Start” is the initial position of the AUV, “Target” is the initial position of the target, and “End” is the meeting point of the two, that is, the destination endpoint.

As shown in Figure 14 and Figure 15, under incomplete information conditions, the proposed mission planning method can designa path that meets multiple constraints, avoids threat areas, and encounters the target as early as possible while also providing the angle at which the path enters the target dispersion area. When the AUV’s movement azimuth is 3.62 radians, the resulting enclosing circle is the smallest, and the AUV can reach the target’s minimum enclosing circle within 97.04 s, which is the shortest time obtained. The difference between this time and the best given time is only 0.09 s, ensuring that the AUV can encounter the dynamic target in a timely manner.

As can be seen from Table 7, due to the influence of the initial target information, the planning accuracy under complete or incomplete information conditions is not significant. In the complete information scenario, the maximum deviation rate of planning time decreases by approximately 6.9%, while in the incomplete information scenario, the average deviation rate of planning time decreases by approximately 7.8%.

## 4. Conclusions

In general, the task planning problem for AUVs to pursue dynamic targets can be divided into two parts: whether the AUV can encounter the dynamic target and whether the encounter time is the shortest. The mutual coupling of these two problems makes their resolution difficult. This paper proposes an AUV autonomous global task planning method by combining two typical initial observation target information scenarios. By integrating the idea of hierarchical planning with the snake optimization algorithm, the global task planning of the AUV achieves the shortest encounter time with the dynamic target. Through mathematical simulations, it is verified that the hierarchical mission planning methods proposed in this article can effectively solve the AUV global mission planning problem, enabling the AUV to encounter dynamic targets in the shortest time possible under multiple constraints.

This study innovatively adopts a three-layer planning strategy to address the two mutually coupled problems of the AUV encountering the dynamic target and minimizing the encounter time. This enables the algorithm to obtain a global optimal solution for dynamic targets. Additionally, the novel snake optimization algorithm is introduced into the task planning of each layer, and two different planning methods are proposed based on two typical initial detection target information of the platform, allowing the algorithm to address issues related to dynamic targets and effectively solve practical engineering problems.

This study addresses the issue of underwater autonomous task planning for AUVs, providing a method for autonomous unmanned underwater vehicle task planning. Focusing on the research of methods, some simplifications are made to the environment in this study. For example, the influence of the AUV’s own volume is ignored, and a fixed flight speed is set. In subsequent research, we will consider the influence of the vehicle’s own factors and explore the AUV’s autonomous task planning capabilities under non-uniform speed conditions.

This article proposes a hierarchical task planning method based on the snake optimization algorithm for AUV task allocation and path planning problems. Considering the coupling relationship between task allocation and path planning, the AUV task planning problem wasbroken downinto multiple nested optimization design problems through hierarchical partitioning and solved using the snake optimization algorithm. This algorithm can effectively solve the task planning problem of AUV under different initial information conditions, providing new ideas and methods for the practical application of AUV. In future work, research should focus on improving the algorithm, enhancing its ability to adapt to complex dynamic environments, and expanding its application scenarios, such as considering the collaboration and heterogeneity of multiple drones and introducing parallel computing mechanisms to improve design efficiency.

## Figures and Tables

**Table 1:** Obstacle parameters.

x	y	z	R
(m)	(m)	(m)	(m)
350	500	100	60
600	200	150	70
500	350	150	80
350	200	150	70
700	550	150	70
650	750	150	80
800	400	150	70
300	350	100	50
500	600	100	50

**Table 2:** SO Algorithm Parameter Definition.

Symbol	Meaning
Am, Af	Represent the ability of males and females to find things, respectively
rand	Represents a random number within the range [0,1]
N, Nm, Nf	Represent the snake population size, the number of males, and the number of females, respectively
t, T	Represent the current iteration number and the maxi-mum number of iterations, respectively
c1, c2 and c3	Are all constants, respectively, 0.5, 0.05, and 2
Q	Food quantity
Temp	Temperature
FF, FM	Represent the fighting ability of males and females, respectively
*X*_*i*,max_, *X*_*i*,min_	Solve the upper and lower boundaries of the problem
Limits	Solve the difference between the upper and lower boundaries of the problem, that is, Xmax–Xmin
*X*_*i*,*m*_, *X*_*i*,*f*_, *X*_*i*_	Represent the position of the ith individual in males, females, and the entire population, respectively
f_*i*,*m*_, f_*i*,*f*_, f_*i*_	Represent the fitness of the ith individual in males, females, and the entire population, respectively
X_*best*,*m*_, X_*best*,*f*_ and X_*food*_	Represent the position of the best individual in males, females, and the entire population, respectively
*f*_*best*,*m*_, *f*_*best*,*f*_ and *f*_*food*_	Represent the fitness of the best individual in males, females, and the entire population, respectively
X_rand,m_, X_rand,f_	Random positions of males and females
f_rand,m_, f_rand,f_	Random positions of males and fitness of females
X_worst,m_, X_worst,f_	Represent the position of the worst individual in males and females, respectively.

**Table 3:** Task planning input parameters under the complete information scenario.

Section	Parameter	Value
Initialization parameters	AUV initial position	[200,100,150] m
	Target initial position	[900,700,100] m
	AUV speed	10 m/s
	Target speed	0.5 m/s
	Initial given time	200 s
	Path node count	15
Internal optimization algorithm parameters	Internal snake optimization algorithm population size	50
	Internal snake optimization algorithm iteration count	100
	Food judgment value	0.25
	Temperature judgment value	0.6
	C1	0.5
	C2	0.05
	C3	2
Intermediate optimization algorithm parameters	Intermediate snake optimization algorithm population size	20
	Intermediate snake optimization algorithm iteration count	5
	Food judgment value	0.25
	Temperature judgment value	0.6
	C1	0.5
	C2	0.05
	C3	2
External optimization algorithm parameters	External snake optimization algorithm population size	20
	External snake optimization algorithm iteration count	5
	Food judgment value	0.25
	Temperature judgment value	0.6
	C1	0.5
	C2	0.05
	C3	2

**Table 4:** Simulation results under complete information conditions.

Count	Best_Time	Practical_Time	Delat_Time	Voyage
	(s)	(s)	(s)	(m)
1	102.3396	98.8857	3.4539	998.857
2	98.8897	98.1893	0.7004	981.893
3	102.4319	99.8661	2.5658	998.661
4	101.9350	101.3425	0.5942	1013.425
5	100.8928	100.9090	2.9838	1009.090
6	100.8224	100.7205	0.1018	1007.205
7	102.3045	101.3076	0.9969	1013.076
8	96.5964	95.4761	1.1204	954.761
9	101.6371	100.7935	0.8437	1007.935
10	94.8353	94.0722	0.7631	940.722
MAX	102.4319	101.3425	3.4539	1013.425
MIN	94.8353	94.0722	0.1018	940.722
MEAN	100.26847	99.15625	1.4124	992.5625

**Table 5:** Task planning input parameters under theincomplete information scenario.

Section	Parameter	Value
Initialization Parameters	AUV initial position	[200,100,150] m
	Target initial position	[900,700,100] m
	AUV speed	10 m/s
	target speed	0.5 m/s
	Initial given time	200 s
	Path node count	pi
Internal optimization algorithm parameters	Internal snake optimization algorithm population size	50
	Internal snake optimization algorithm iteration count	100
	Food judgment value	0.25
	Temperature judgment value	0.6
	C1	0.5
	C2	0.05
	C3	2
Intermediate optimization algorithm parameters	Intermediate snake optimization algorithm population size	20
	Intermediate snake optimization algorithm iteration count	5
	Food judgment value	0.25
	Temperature judgment value	0.6
	C1	0.5
	C2	0.05
	C3	2
External optimization algorithm parameters	External snake optimization algorithm population size	20
	External snake optimization algorithm iteration count	5
	Food judgment value	0.25
	temperature judgment value	0.6
	C1	0.5
	C2	0.05
	C3	2

**Table 6:** Simulation results under incomplete information conditions.

Count	Best_Time	Practical_Time	Delat_Time	Voyage	Best_Theta
	(s)	(s)	(s)	(m)	(rad)
1	109.50	110.15	0.64	1101.46	3.69
2	112.96	111.91	1.052	1119.11	6.28
3	100.31	104.15	3.84	1041.45	4.16
4	106.98	107.53	0.55	1075.31	1.62
5	103.79	103.69	0.09	1036.94	1.33
6	99.45	99.73	0.29	997.31	2.34
7	101.17	100.78	0.38	1007.85	2.99
8	100.14	97.75	2.40	977.46	3.89
9	97.44	100.94	3.50	1009.46	4.28
10	97.84	97.04	0.8	970.37	3.62
MAX	112.96	111.91	3.84	1119.11	6.28
MIN	97.44	97.04	0.09	970.37	1.33
MEAN	103.87	104.14	1.58	1041.44	3.81

**Table 7:** Comparison of planning performance under different information conditions.

Initial Information Conditions	Maximum Deviation Rate	Minimum Deviation Rate	Average Deviation Rate
Complete information	3.37%	0.1%	1.4%
Incomplete information	3.6%	0.09%	1.52%

## Data Availability

Data are contained within the article.
